# Proteomic analysis of corneal endothelial cell-descemet membrane tissues reveals influence of insulin dependence and disease severity in type 2 diabetes mellitus

**DOI:** 10.1371/journal.pone.0192287

**Published:** 2018-03-12

**Authors:** Jessica M. Skeie, Benjamin T. Aldrich, Andrew S. Goldstein, Gregory A. Schmidt, Cynthia R. Reed, Mark A. Greiner

**Affiliations:** 1 University of Iowa Carver College of Medicine, Department of Ophthalmology and Visual Sciences, Iowa City, United States of America; 2 Iowa Lions Eye Bank, Coralville, United States of America; 3 Cornea Research Center, University of Iowa, Iowa City, United States of America; Cedars-Sinai Medical Center, UNITED STATES

## Abstract

The objective of this study was to characterize the proteome of the corneal endothelial cell layer and its basement membrane (Descemet membrane) in humans with various severities of type II diabetes mellitus compared to controls, and identify differentially expressed proteins across a range of diabetic disease severities that may influence corneal endothelial cell health. Endothelium-Descemet membrane complex tissues were peeled from transplant suitable donor corneas. Protein fractions were isolated from each sample and subjected to multidimensional liquid chromatography and tandem mass spectrometry. Peptide spectra were matched to the human proteome, assigned gene ontology, and grouped into protein signaling pathways unique to each of the disease states. We identified an average of 12,472 unique proteins in each of the endothelium-Descemet membrane complex tissue samples. There were 2,409 differentially expressed protein isoforms that included previously known risk factors for type II diabetes mellitus related to metabolic processes, oxidative stress, and inflammation. Gene ontology analysis demonstrated that diabetes progression has many protein footprints related to metabolic processes, binding, and catalysis. The most represented pathways involved in diabetes progression included mitochondrial dysfunction, cell-cell junction structure, and protein synthesis regulation. This proteomic dataset identifies novel corneal endothelial cell and Descemet membrane protein expression in various stages of diabetic disease. These findings give insight into the mechanisms involved in diabetes progression relevant to the corneal endothelium and its basement membrane, prioritize new pathways for therapeutic targeting, and provide insight into potential biomarkers for determining the health of this tissue.

## Introduction

**Fig 1:**
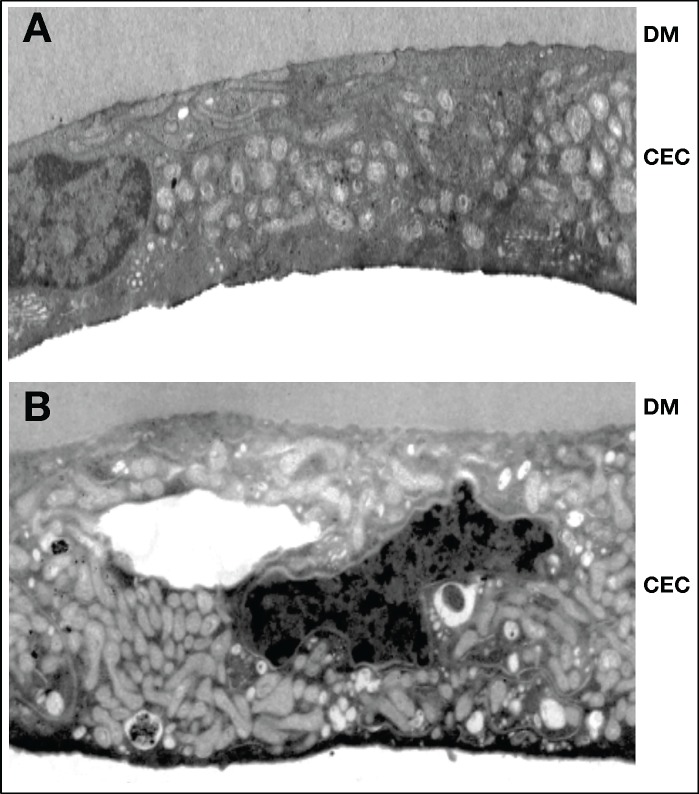
Corneal endothelial cell morphology. Corneal endothelial cells without diabetes (A; control) and with diabetes (B). The cells in diabetic corneas are distended and have altered appearing mitochondria, dark inclusion bodies, a dark apical surface, a greater number of vacuole type bodies, and altered golgi compared to control tissues. The dark appearance of the inclusion bodies–within organelles and the cytosol, as well as on the apical surface–indicates aggregates of possible protein and lipid waste. Mitochondrial and golgi alterations were consistent among all affected samples and indicate potential changes in organelle dynamics in disease states. DM, Descemet membrane; CEC, corneal endothelial cell.

Corneal endothelial cells and their basement membrane (Descemet membrane) are impaired functionally [1–10], morphologically [11, 12], and biochemically [13–17] by diabetes mellitus. This has been demonstrated in the literature with increased endothelial cell loss after cateract surgery [2, 3, 18] and worse outcomes after Descemet membrane endothelial keratoplasty (DMEK) [19]. It is known that the endothelial cell density is decreased in donor corneas with type 2 diabetes mellitus (T2DM) [20–23], indicating possible cell death and dropout due to disease. Our group has shown that separation of the corneal endothelium-Descemet membrane complex (EDM) from the underlying stroma in corneas with T2DM requires greater force compared to nondiabetic tissues [24], indicating that the basement membrane itself is structurally altered due to disease. Additionally, we have shown that mitochondrial function is impaired and morphologically altered (Fig 1) in donor corneas with T2DM [25], indicating an increase in mitochondrial dysfunction due to disease. Although we have shown deficiencies in donor EDM tissues due to T2DM [26], the process by which the disease causes these changes is still unknown [27].

Proteomics has been used widely for understanding disease pathogenesis [28], but its adoption into studies of ocular health and disease is a more recent occurrence [29]. In diabetes mellitus, tissues such as skeletal muscle have been studied with proteomics surveys, and several cellular proteome shifts have been linked to changes in skeletal muscle cell function and morphology. Specifically, proteomic changes indicative of oxidative phosphorylation [30–33], insulin resistance [34], mitochondrial dynamics [35–38], oxidative stress [39, 40], and inflammation [41–43] have been characterized. The ophthalmology literature has discussed the value of proteomic analysis for identifying disease biomarkers [44], but this approach has not been applied widely. A demonstrated use for ocular proteomics in diabetes was shown when pre-retinopathic biomarkers were identified in diabetic retinal pigment epithelium [45]. Although the corneal proteome has been identified [46], to the best of our knowledge the effects of diabetes on the corneal endothelium have not been studied using this technique.

We anticipate that key protein signals altered by T2DM in other tissues will also be affected in corneal EDM tissues. To investigate this hypothesis, we mapped the entire proteome of donor EDM tissues from individuals with various severities of T2DM and compared them to proteomes of individuals without disease. This complete set of data serves as a resource for determining statistically significant changes marked by disease progression, as well as for future inquiries related to diabetic effects on corneal endothelial cells. These data will support future efforts to determine accurate biomarkers for assessing the health of donor corneas and the effecacy of therapeutics to prevent vision loss, in addition to inquiries about the effects of diabetes on corneal endothelial cells in general.

## Materials and methods

This study was approved by the University of Iowa’s Institutional Review Board and adhered to the tenants of the Declaration of Helsinki. None of the tissue donors from whom samples were procured for this investigation were from a vulnerable population, and all donors or next of kin provided appropriate consent for tissue donation and research.

### Human tissue sample collection and grouping

EDM tissue samples were prepared from cornea donors with and without T2DM and were classified into different groups based on severity as published previously [25, 47]. Samples from donors lacking a diagnosis of diabetes mellitus or complications due to diabetes (diabetic neuropathy, diabetic retinopathy, renal failure due to diabetes, amputations due to diabetes) in their medical history were defined as the ***nondiabetic control (ND) sample group*.** Samples from diabetic donors with no notation of medical complications secondary to diabetes (*without [-ni] or with [-i] history of home insulin use*) were classified as the ***nonadvanced diabetes (NAD-ni or NAD-i) sample group***. Finally, samples from donors with diabetes mellitus that had a *history of home insulin use* and end-organ damage specifically noted in the medical history as occurring as a result of diabetes were classified as the ***advanced diabetes (AD) sample group***. Myocardial infarction was not considered a complication specific to diabetes for any of these groupings due to the high incidence of comorbidity in the US population.

Donor corneas were preserved at 4°C in storage media at (Optisol GS, Bausch & Lomb, Irvine, CA) within 24 hours of death and assayed within 14 days of preservation from donors aged 50–75 years. Corneas were evaluated by qualified eye bank staff using Eye Bank Association of America and Iowa Lions Eye Bank standard protocols and procedures. To prepare EDM tissues, corneas were mounted onto a 9.5 mm vacuum trephine (Barron Precision Instruments, LLC, Grand Blanc, MI) and scored through the EDM into the sclera. EDM tissues were visualized using VisionBlue (DORC International, Zuidland, The Netherlands) and carefully peeled away from the stroma. Samples were put into 1.5 mL Eppendorf tubes and stored immediately in a -80°C freezer.

### Multidimensional protein identification technology (MudPIT) mass spectrometry

EDM tissue samples were prepared for digestion using the filter-assisted sample preparation (FASP) method [48]. Samples were first digested in 5% sodium deoxycholate (SDC), 100 mM triethylammonium bicarbonate (TEAB), pH 8.0, 3 mM dithiothreitol (DTT), sonicated, and spun down, and then the supernatant was transferred to a 30 kD MWCO device (Millipore; Billerica, MA) and centrifuged at 13,000 g for 30 minutes. The remaining sample was buffer exchanged with 1% SDC, 100 mM TEAB, pH 8.0, then alkylated with 15 mM iodoacetamide before being digested overnight at 37°C using trypsin at an enzyme to substrate ratio of 1:100 in a Thermo-Mixer (Thermo Fisher Scientific; Waltham, MA) at 1000 RPM. Digested peptides were collected by centrifugation. Approximately 20 μg of peptides were desalted using reversed phase stop-and-go extraction (STAGE) tips [49], eluted with 80% acetonitrile, 5% ammonium hydroxide and lyophilized in a SpeedVac (Thermo Fisher Scientific; Waltham, MA) for 1 hour.

Each peptide sample was analyzed by ultra-high performance liquid chromatography tandem mass spectrometry (UHPLC-MS/MS). Liquid chromatography (LC) was performed on an Easy-nLC 1000 UHPLC system (Thermo Fisher Scientific) with mobile phase A composed of 97.5% MilliQ water, 2% acetonitrile, and 0.5% formic acid, and mobile phase B composed of 99.5% acetonitrile, and 0.5% formic acid. The 240 minute LC gradient ran from 0% B to 25% B over 210 minutes, then to 80% B for the remaining 30 minutes. Samples were loaded directly to the column (50 cm x 75 μm inner diameter [I.D.]) and packed with 2 μm C18 media (Thermo Easy Spray PepMap; Thermo Fisher Scientific). The LC was interfaced to a quadrupole-Orbitrap mass spectrometer (Q-Exactive; Thermo Fisher Scientific) via nano-electrospray ionization (Thermo Easy Spray source; Thermo Fisher Scientific) and heated to 50°C with an electrospray voltage of 2.2 kV. The mass spectrometer was programmed to acquire tandem mass spectra from the top 20 ions in the full scan from 400–1200 m/z. Singly-charged ions were excluded and dynamic exclusion was set to 15 seconds, isolation width to 1.6 Dalton, full MS resolution to 70,000 and MS/MS resolution to 17,500. Normalized collision energy was set to 25 eV, automatic gain control to 2e5, max fill MS to 20 milliseconds, max fill MS/MS to 60 milliseconds, and the underfill ratio to 0.1%.

In order to identify peptides, mass spectrometer RAW data files were converted to mzML format using msconvert [50], then MGF files were generated from mzML using the Peak Picker HiRes tool from the OpenMS framework [51]. All searches required 10 parts per million precursor mass tolerance, 0.02 Dalton fragment mass tolerance, strict tryptic cleavage, up to 2 missed cleavages, fixed modification of cysteine alkylation, variable modification of methionine oxidation, and protein-level expectation value scores of 0.0001 or lower. MGF files were searched using the most recent protein sequence libraries available from UniProtKB, X!Tandem [52], and OMSSA [53].

### Bioinformatics and statistical analysis

A systems biology approach was implemented using several bioinformatics analyses to determine significant protein expression (Partek Geonomics Suite 6.6; Partek Inc., St. Louis, MO), gene ontology (GO terminology, Panther 7.2) [54], and pathway representation (Ingenuity Pathway Analysis, IPA; Qiagen, Germantown, MD). Protein intensities were scaled to logarithmic base 10, and statistically significant proteins (analysis of variance [ANOVA], P < 0.05) were visualized using an undiscriminated clustered heatmap with a normalized clustering function, creating map patterns based only on clustering of protein expression pattern similarities. Networks of associated proteins were created using String database v10.5 [55]. Canonical pathway representation and significance was determined by the number of proteins in the dataset in common with known proteins in a single pathway. Proteins that showed a trend but did not meet statistical significance (P < 0.05) underwent further bioinformatics analysis if they were an important component of a specific pathway or classification.

## Results

EDM samples were collected from 19 donors (4 from nondiabetic controls, 10 with nonadvanced diabetes [5 non-insulin dependant, 5 insulin dependant], and 5 with advanced diabetes; Table 1). The average age for donors was 65.5 years (standard deviation, 2.0 years) and average death to preservation time for all samples was 9.35 days (standard deviation, 2.81 days). The body mass index (BMI) averages for each group were 22.2 in the ND group, 30.8 in the NAD-ni group, 46.0 in the NAD-i group, and 26.9 in the AD group.

**Table 1:** Donor EDM sample demographics.

#	Diagnosis	Age	Sex	BMI	ECD	Medical History
1	ND	58	M	29.7	2,387	ASCVD
2	ND	73	M	26.3	2,326	respiratory failure, COPD, AAA, heart arrhythmia, hypoxemia, eczema, diverticular disease, sleep apnea, osteoporosis, left ventricular hypertrophy, chronic back pain, night sweats, fluid retention
3	ND	60	F	12.4	2,336	cardiopulmonary arrest, lung cancer, pneumonia, COPD, emphysema, electrolyte imbalance, malnutrition, joint pain, rash, night sweats, joint pain, back pain, leg pain
4	ND	66	M	20.4	1,779	respiratory failure, CHF, COPD, pneumonia, hyperlipidemia, HTN, MI
5	NAD-ni	67	F	45.7	1,043	cardiopulmonary arrest, HTN, COPD, hyperlipidemia, renal failure, small vessel ischemia, morbid obesity, hematuria, arthritis, chronic edema, sleep apnea, MI, right leg swelling, hyperparathyroidism, cardiac catheritization, CHF
6	NAD-ni	59	M	28.8	2,358	MI, hypothyroidism, hyperlipidemia, renal insufficiency, obesity, high cholesterol, lumbar disc degeneration, ear infection, blue toes, toe cyanosis, abscessed tooth, allergies, sebasceous cyst, benign skin lesion
7	NAD-ni	74	F	14.7	2,519	heart failure, COPD, ischemic cardiomyopathy, diverticulitis, weight loss, stroke, leg joint pain, anemia, CAD, HTN, dyslipidemia, difficulty walking
8	NAD-ni	67	M	34.6	1,972	cardiopulmonary arrest, hypoglycemia, CHF, obesity, liver & kidney insufficiency, clotting disorder, skin sores, diarrhea, cough, COPD, hypercholesterolemia, HTN, sleep apnea, hemorrhoids, pneumonia, CRF, hypoglycemia, hepatocellular liver damage, pain, muscle spasms, SOB, wheezing
9	NAD-ni	61	F	30.1	2,208	anxiety, asthma, HTN, COPD, CAD, obesity
10	NAD-i	71	F	40.8	2,564	cardiopulmonary arrest, cancer, CAD, CHF, CKD, kidney failure, HTN, hypothyroidism, diaphoresis, chest pain, elevated troponins, SOB, multivessel disease, hyponatremia, MI, morbid obesity, sleep apnea, stroke, COPD, anemia, hyperlipidemia, carpal tunnel, arthritis, night sweats, fatty tumor in leg
11	NAD-i	65	F	29.2	2,049	CHF, hypokalemia, neuropathy, hypoglycemia, hypokalemia, HTN, MRSA
12	NAD-i	73	M	44.5	1,429	cardiopulmonary arrest, morbid obesity, hyperlipidemia, gout, hypertension, double vision headaches, difficulty walking, HTN, skin discoloration on front of legs, sleep apnea, latex allergy, hyperkalemia, anemia, CKD
13	NAD-i	66	M	63.8	2,445	chronic respiratory failure, pneumonia, neuropathy, morbid obesity, CHF, respiratory failure, pulmonary HTN, COPD, OSA, cirrhosis, arthritis, obesity hypoventilation syndrome
14	NAD-i	66	M	51.6	2,398	CVA, HTN, neuropathy, hyperlipidemia, cerebrovascular disease, CKD, GERD, depression
15	AD	68	M	22.7	2,841	cardiopulmonary arrest, CHF, COPD, UTI, diabetic polyneuropathy, vascular disease, CAD, hyperlimidemia, pulmonary disease, supraventricular arrhythma, venous disease, pain, non-healing elbow skin lesion, skin disorder, tremors, muscle spasms, diverticulosis, colon polyp, hemorrhoids, ischemis colitis, hernia, rectal abscess, difficulty walking, shoulder impingment, groin abscess, basal cell cancer, kidney stones, legs turn blue when standing, anxiety, BPH, chronic pain syndrome, dermatitis, dysphagia, fatigue, GERD, back pain, ED, arthritis, insomnia, tinnitus, urinary retention, vertigo, thyroid disorder
16	AD	65	M	22.5	2,278	CHF, CAD, ischemic heart failure, hyperlipidemia, HTN, CVA, OSA, obesity, DVT, diabetic neuropathy, diabetic retinopathy, COPD
17	AD	69	F	19.4	1,447	head bleed, HTN, diabetic peripheral neuropathy, COPD, amputation, diabetic CKD, anxiety, depression, mitral valve disease, restless leg syndrome, PVD, carpal tunnel, bowel obstruction, glaucoma, vitrectomy
18	AD	62	M	28.8	2,016	ASCVD, diabetic vascular disease, chronic gout of multiple sites, neuropathy, asthma, gastric ulcer, diverticulitis, arthritis, osteoporosis, overweight, oxygen dependence, MRSA, COPD, HTN, chronic anticoagulation, stage III diabetic CKD, OSA, chronic hypoxemic respiratory failure, insomnia, hyperlipidemia, CHF, heart attack, GERD, barrett esophagus, chronic pain syndrome, anemia, benign lung tumor, pneumonia, diabetic sores, night sweats, leg swelling, difficulty walking
19	AD	55	F	41.2	2,137	ASCVD, CAD, hyperlipidemia, diabetic neuropathy, vulvar cancer, coronary atherosclerosis, MI, HTN, GERD, OSA, hypothyroidism, RA, fibromyalgia, depression, anxiety, bipolar disorder

Abbreviations: AAA, abdominal aortic aneurysm; AD, advanced diabetes; ASCVD, atherosclerotic cardiovascular disease; BMI, body mass index (kg/m^2^); BPH, benign prostatic hyperplasia; CAD; coronary artery disease; CHF, congestive heart failure; CKD, chronic kidney disease; COPD, chronic obstructive pulmonary disease; CRF, chronic renal failure; CVA, cerebrovascular accident; DVT, deep vein thrombosis; ECD, endothelial cell density (cells/mm^2^); ED, erectile dysfunction; GERD, gastroesophageal reflex disease; HTN, hypertension; MI, myocardial infarction; MRSA, methicillin-resistant *Staphlococcus aureus*; NAD, nonadvanced diabetes ([n]i, [no] insulin); ND, nondiabetic control; OSA, obstructive sleep apnea; PVA, peripheral vascular disease; RA, rheumatoid arthritis; SOB, shortness of breath; UTI, urinary tract infection.

### Mass spectrometry overview

EDM samples underwent trypsinization and multidimensional liquid chromatography before analysis by tandem mass spectrometry. In the 19 samples included for analysis, we identified an average of 146,979 spectra (31,693 unique peptides) per sample, and an average of 12,472 unique proteins (25,898 unique proteins including all isoforms) per sample. The most abundant proteins identified overall were transforming growth factor-beta-induced (TGFBI), alpha-enolase (ENO1), c-myc promotor binding protein 1, glyceraldehyde-3-phosphate dehydrogenase (GAPDH), pyruvate kinase PKM (PKM), L-lactate dehydrogenase A chain (LDHA), basement membrane specific heparin sulfate proteoglycan core protein (HSPG2), fructose-bisphosphate aldolase A (ALDOA), beta actin variant, and cytoplasmic actin 2 (ACTG1). Full protein datasets are available as supplemental data (S1 Table).

### ANOVA analysis and clustered heatmapping

**Fig 2:**
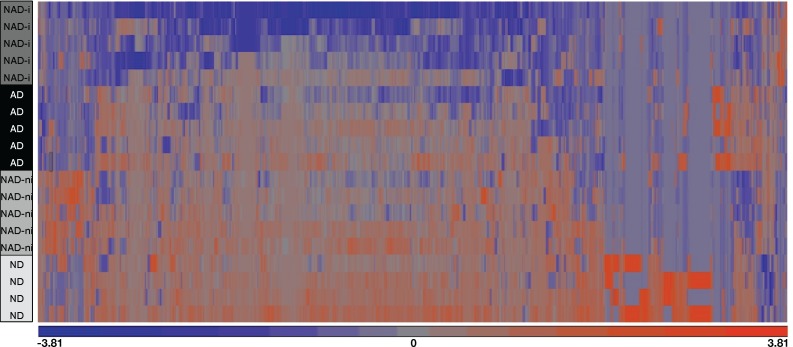
Differential protein expression. Several proteins are differentially expressed in the EDM tissues of patients with different severities of diabetes mellitus. Unbiased clustering of proteins differentially expressed in the four different severities of disease used in this study. Proteins represented in this cluster analysis were determined by ANOVA, P < 0.05. Orange indicates protein upregulation, purple/blue indicates downregulation, and gray represents no expression.

Following ANOVA statistical analysis of non-normalized proteins, comparing the four disease severity groups (ND, NAD-ni, NAD-i, and AD), we detected 2,409 protein isoforms with statistically significant changes (S2 Table; P < 0.05). These proteins were clustered using a non-biased algorithm and several unique sets of proteins in each group distinguished each stage of disease severity as unique from each other and consistent among donors (Fig 2).

### Gene ontology

**Fig 3:**
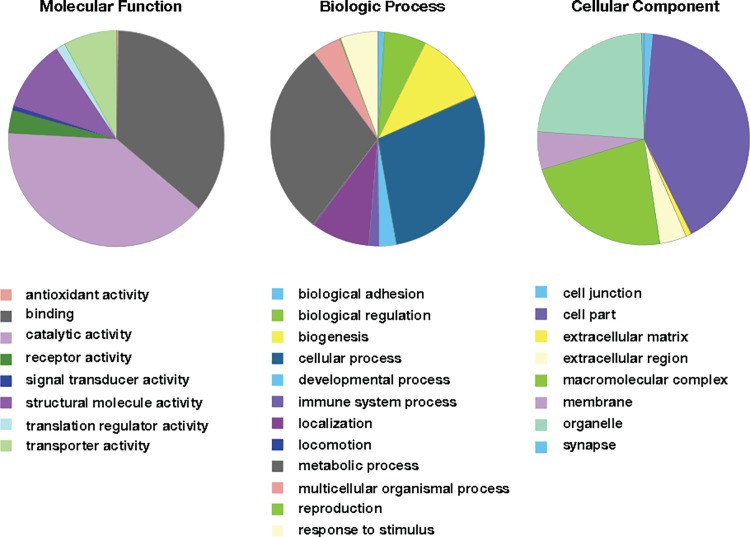
Gene ontology (GO) distributions of differentially expressed EDM tissue proteins in increasing severity of diabetes mellitus. Differentially expressed proteins were determined by ANOVA analysis (P < 0.05). Proteins were grouped into sub-categories of biological processes, molecular functions, and cellular component.

After identifying statistically significant proteins that were expressed differentially among the four disease severity groups, we categorized the proteins using gene ontology groupings. For molecular function, differentially expressed proteins (including all isoforms) were mostly involved in binding and catalytic activity. For biological processes, differentially expressed proteins were mostly involved in metabolic process, cellular process, and biogenesis. For the cellular component category, differentially expressed proteins were mostly from cell part, organelle, and macromolecular complexes. A complete listing of differentially expressed proteins by ontological grouping can be found in Fig 3.

### Molecular pathways

**Fig 4:**
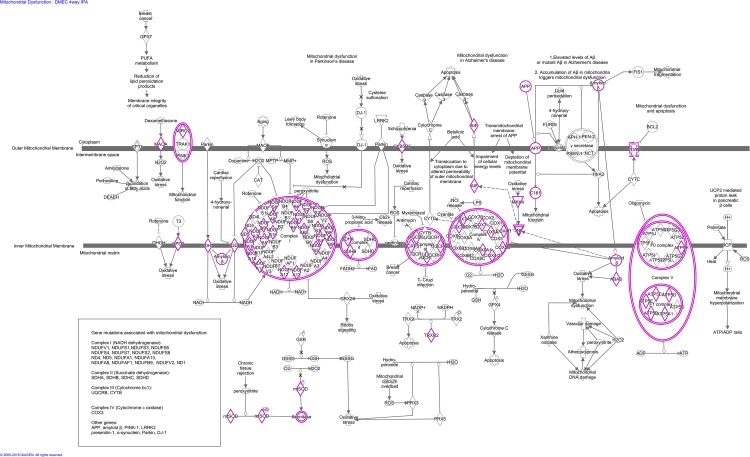
Overview diagram of differentially expressed human EDM proteins in increasing severity of diabetes mellitus involved in oxidative phosphorylation. All proteins in the diagram highlighted in pink were altered statistically relative to increasing disease severity. Oxidative phosphorylation and mitochondrial dysfunction were the two most affected cellular mechanisms during the progression of diabetes mellitus type II in EDM tissues. Figure were created using IPA (Qiagen, Germantown, MD).

A molecular pathway analysis of the statistically significant proteins expressed differentially by disease severity identified several groups of functionally related proteins. It is important to note that pathways represented by a dataset may have names representative of a disease that they are most commonly affiliated with or by which they were discovered, and that these names are not necessarily indicative of the mechanism responsible for the protein footprint found in a particular tissue. The top ten global pathways related to diabetes mellitus progression in the EDM tissues were mitochondrial dysfunction (Table 2), oxidative phosphorylation (Fig 4), eukaryotic initiation factor 2 (eIF2) signaling (Table 3), regulation of eukaryotic initiation factor 4 (eIF4) and ribosomal protein S6 kinase (p70S6K) signaling, mechanistic target of rapamycin kinase (mTOR) signaling (Table 4), Sertoli cell-Sertoli cell junction signaling, cyclin dependant kinase 5 (CDK5) signaling, integrin linked kinase (ILK) signaling, role of janus kinase (JAK) family kinases in interleukin 6 (IL-6) type cytokine signaling, and UVB induced mitogen-activated protein kinase (MAPK) signaling. A complete listing of differentially expressed proteins by molecular pathway can be found in S3 Table.

**Table 2:** Top 12 most significantly altered proteins in T2DM in EDM tissues associated with mitochondrial dysfunction.

Gene ID	UniProt ID	NAD-ni v ND	NAD-I v ND	AD v ND	NAD-i v NAD-ni	AD v NAD-i	AD v NAD-ni	p-value
*NDUFA5*	A0A087WXR5	-1.05	-1.57	-1.11	-1.49	1.41	-1.06	0.0013
*NDUFS8*	F8W9K7	-1.10	-1.50	-1.16	-1.36	1.30	-1.05	0.0033
*ATP5D*	P30049	-1.06	-1.60	-1.12	-1.51	1.43	-1.06	0.0043
*ATP5C1*	Q8TAS0	-1.09	-1.60	-1.17	-1.47	1.37	-1.07	0.0048
*NDUFA8*	B7Z768	-1.06	-1.61	-1.18	-1.52	1.37	-1.11	0.0049
*SDHB*	P21912	-1.12	-1.54	-1.14	-1.38	1.35	-1.02	0.0050
*NDUFA11*	K7ELJ0	-1.18	-1.75	-1.47	-1.49	1.19	-1.25	0.0054
*COX5A*	Q71UP1	1.03	-2.01	-1.10	-2.08	1.82	-1.14	0.0058
*NDUFB5*	B2R9L0	-1.15	-1.92	-1.14	-1.67	1.69	1.01	0.0061
*UQCRQ*	B7Z4A1	-1.21	-1.66	-1.38	-1.37	1.21	-1.14	0.0061
*COX7C*	A0A024RAP6	-1.17	-1.53	1.18	-1.31	1.80	1.38	0.0063
*NDUFA7*	M0R1K9	1.01	-2.42	1.00	-2.44	2.43	-1.00	0.0063

Fold changes are given for all individual comparisons. P-values are given for 4-way ANOVA analysis while all available 2-way p-values can be found in S2 Table. Proteins were determined using IPA (Qiagen, Germantown, MD).

**Table 3:** Top 12 most significantly altered proteins in T2DM in EDM tissues associated with eIF2 signaling.

Gene ID	UniProt ID	NAD-ni v ND	NAD-I v ND	AD v ND	NAD-i v NAD-ni	AD v NAD-i	AD v NAD-ni	p-value
*RPL3L*	Q92901	9.04	1.00	203.52	-9.04	203.52	22.51	0.0002
*RPS14*	E5RH77	-1.33	-1.09	-1.17	-1.22	1.14	-1.07	0.0033
*RPS23*	P62266	-1.25	-1.72	-1.38	-1.38	1.25	-1.10	0.0038
*RPL15*	E7ERA2	-1.03	-83.88	-3.08	-81.36	27.20	-2.99	0.0045
*RPL23A*	A8MUS3	-1.21	-110.26	-3.41	-91.38	32.35	-2.83	0.0049
*EIF3K*	U3LUI4	-1.20	-1.77	-1.29	-1.47	1.37	-1.07	0.0050
*RPL4*	H3BU31	-1.07	-2.42	-1.16	-2.27	2.09	-1.09	0.0051
*RPL7*	A0A024R814	-1.10	-2.94	-1.35	-2.68	2.18	-1.23	0.0053
*RPL32*	D3YTB1	-1.60	-1.52	-1.19	1.06	1.27	1.34	0.0056
*EIF4E*	B7Z2T1	1.00	18.75	51.90	18.75	2.77	51.90	0.0067
*RPL10A*	Q1JQ76	-1.07	-1.62	-1.29	-1.51	1.26	-1.20	0.0069
*RPL3*	B5MCW2	1.05	-2.07	-1.08	-2.17	1.92	-1.13	0.0070

Fold changes are given for all individual comparisons. P-values are given for 4-way ANOVA analysis while all available 2-way p-values can be found in S2 Table. Proteins were determined using IPA (Qiagen, Germantown, MD).

**Table 4:** Top 12 most significantly altered proteins in T2DM in EDM tissues associated with mTOR signaling.

Gene ID	UniProt ID	NAD-ni v ND	NAD-I v ND	AD v ND	NAD-i v NAD-ni	AD v NAD-i	AD v NAD-ni	p-value
*RPS14*	E5RH77	-1.33	-1.09	-1.17	-1.22	1.14	-1.07	0.0033
*RPS23*	P62266	-1.25	-1.72	-1.38	-1.38	1.25	-1.10	0.0038
*EIF3K*	U3LUI4	-1.20	-1.77	-1.29	-1.47	1.37	-1.07	0.0050
*EIF4E*	B7Z2T1	1.00	18.75	51.90	18.75	2.77	51.90	0.0067
*PTPN11*	Q16344	1.24	-1.61	-1.01	-1.99	1.59	-1.25	0.0081
*RPS16*	Q6IPX4	-1.16	-1.46	-1.15	-1.26	1.27	1.01	0.0096
*RPS2*	Q8J014	1.00	-1.58	-1.07	-1.58	1.47	-1.08	0.0120
*EIF4B*	B4DRM3	-1.01	-1.48	-1.19	-1.46	1.24	-1.18	0.0120
*RPS9*	A5D904	-1.19	-1.60	-1.26	-1.35	1.27	-1.06	0.0120
*PPP2R2B*	G3V149	177.30	7.50	22.24	-23.62	2.96	-7.97	0.0130
*EIF3C*	H3BTY8	-3.76	-91.7	-79.76	-24.39	1.15	-21.22	0.0130
*PPP2R2A*	P63151	69.75	6.94	57.00	-10.05	8.22	-1.22	0.0130

Fold changes are given for all individual comparisons. P-values are given for 4-way ANOVA analysis while all available 2-way p-values can be found in S2 Table. Proteins were determined using IPA (Qiagen, Germantown, MD).

## Discussion

We have uncovered the unique protein profile of the corneal endothelium-Descemet membrane complex (EDM) in human cornea donor tissues suitable for transplantation from nondiabetic controls (ND), nonadvanced diabetics without the use of insulin (NAD-ni), nonadvanced diabetics with the use of insulin (NAD-i), and of advanced diabetics (AD). We have also performed a bioinformatics comparison of these groups to unravel changes in protein profiles during the progression of diabetes and its effects on the posterior cornea.

The analysis considering nondiabetic samples compared to diabetic samples without home insulin use (NAD-ni) revealed that the most significant protein shifts occur in mechanisms involved in glycolysis, transcription, and altering cellular junctions. There were 11 proteins involved directly in glycolysis that had statistically significant changes in individuals still in the early stages of diabetic disease not yet requiring any form of insulin treatment (ALDOA, ALDOC, ENO2, GPI, PFKL, PFKM, PFKP, PGAM1, PGK1, PKM, and TPI1). These findings are consistent with protein changes in glycolytic pathway proteins in other systems. For example, this glycolytic shift has been studied widely as a major contributor to insulin resistance and weakness using proteomics in skeletal muscle [32, 56–58]. We also know that corneal edema is a common finding in diabetic patients [59, 60], which is consistent with poor endothelial cell Na^+^/K^+^-ATPase pump and tight-junction barrier functions secondary to cell health and dropout [1, 61]. Our group has shown previously that endothelial cell density clearly is decreased in donor tissues in the AD group [47]. Our data indicate that there is a proteomic shift in early diabetic disease that reflects alterations in cell to cell junctions, consistent with cells expanding and migrating to reach confluency as cell begin to drop out.

**Fig 5:**
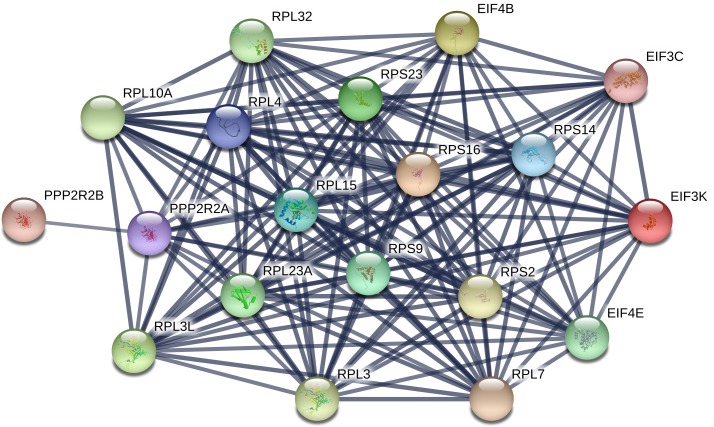
Network diagram of differentially expressed human EDM proteins in increasing severity of diabetes mellitus associated with both eIF2 and mTOR signaling pathways. This network indicates the close relationship of these protein groups. Network created using String database v10.5.

In the analysis comparing any groups containing insulin treatment (all groups, NAD-ni vs. NAD-i, NAD-i vs. AD), the mechanisms altered most significantly were involved in mitochondrial dysfunction, oxidative phosphorylation, translation (eIF2 and eIF4 signaling), stress/apoptosis (mTOR signaling), and cell junctions. eIF2 signaling participates in the unfolded protein response which causes endoplasmic reticulum stress, cell cycle arrest, and/or cellular apoptosis [62]. This mechanism has been shown to play a large role in pancreatic beta cell failure in diabetic patients [63]. mTOR can combine with other proteins to form complexes with different functions (mTORC1 and mTORC2). These complexes regulate cellular functions including but not limited to transcription, survival, autophagy and proliferation, and also participate in insulin signaling [64, 65]. The two complexes work together in a feedback loop to maintain cell growth and metabolism balance [65]. When the formation of mTOR signaling and complex formation is disturbed, the effects contribute to common pathobiologies found in cancer and diabetes including cell stress, apoptosis, metabolic shifts, protein sysnthesis dysregulation, and insulin signaling [66]. These two protein pathways previously have been shown to have many similarities as well as crosstalk [67], a finding corroborated by the overlap in our bioinformatics results (Table 3, Table 4, Fig 5).

The most significantly altered pathway regarding diabetes progression in the EDM tissues was mitochondrial dysfunction. This pathway was not significant, however, when comparing between ND and NAD-ni groups and excluding all groups using insulin therapy. It is noteworthy that the unbiased clustering of the significantly altered proteins in the heatmap (Fig 2) distinguished the NAD-i group away from the NAD-ni group entirely, and closer to AD than to ND. Dendogram groupings and heat map clustering by disease severity were analyzed using Partek software, and these data indicate a strong relationship between these two groups (NAD-i and AD). This relationship is confirmed by our analysis of mitochondria related proteins (S2 Table), which demonstrates a marked decrease in relative protein abundance in insulin dependent samples (NAD-i and AD) compared to non-insulin dependent samples (NAD-ni and ND). Overall, based on molecular pathway analysis, proteins involved in mitochondrial dysfunction seem to incur the greatest reduction as diabetes progresses to insulin dependency, indicating that mitochondrial changes may be related to diabetes insulin therapy itself or disease conditions at the time of transition to insulin therapy. Of note, comparing the NAD-i and AD groups, mitochondria protein levels appear to increase as the disease progresses (Table 2). This finding may indicate a response or reduction in mitochondrial functioning to disease progression and requires further investigation.

**Fig 6:**
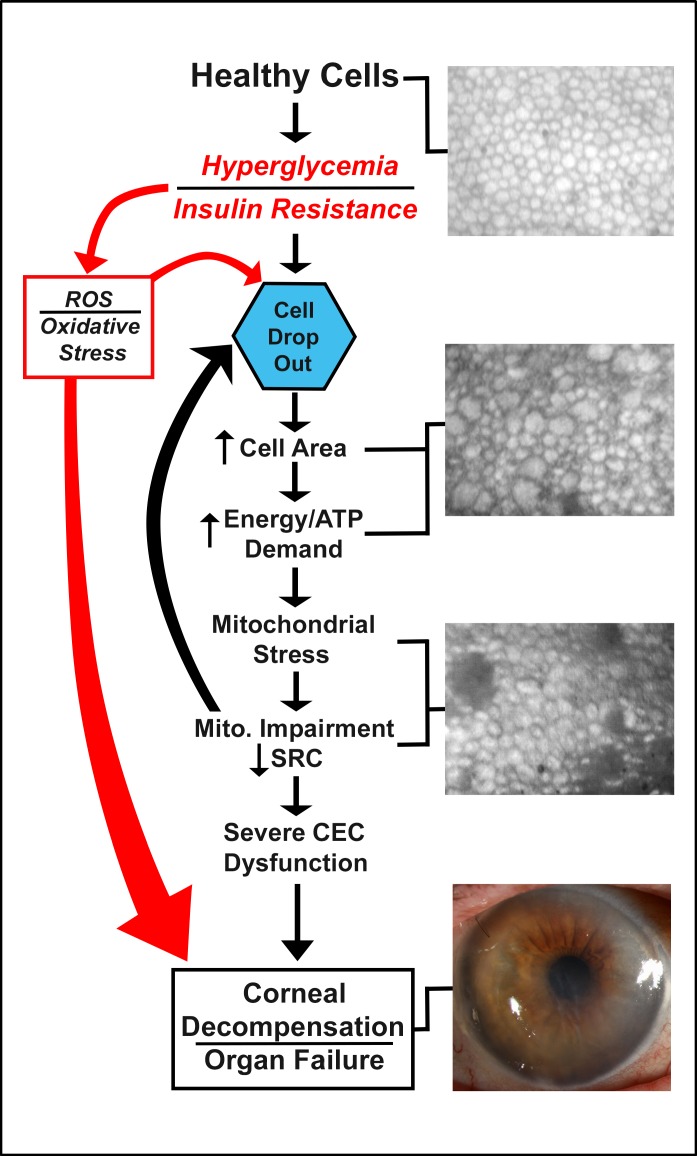
Hypothesis diagram. Hypothesis of how local hyperglycemia affects corneal endothelial cells (CECs) during the progression of T2DM. SRC, spare repiratory capacity as determined using extracellular flux analysis; ROS, reactive oxygen species.

Mitochondrial dysfunction is a general term describing changes in mitochondrial balance including shifts in fission/fusion, mitophagy, redox equilibrium, and inflammation. In the case of insulin resistance, it has been hypothesized that increased demand for energy due to dysfunctional glucose transport causes mitochondrial stress in muscle [68]. Stress on the mitochondria, in turn, results in oxidative stress imbalance and greater amounts of reactive oxygen species detectable within cells [69]. Resultant damage to mDNA, proteins, and lipids further taxes the mitochondria, thus propelling a vicious cycle of mitochondrial dysfunction, low ATP output, increased ATP demands, and insulin resistance [42]. Similarly, our data suggest a hypothesis of impaired corneal endothelial cell health based on mitochondrial dysfunction secondary to chronic hyperglycemia. Our group has previously published the metabolic and morphologic mitochondrial changes found in ex vivo EDM tissues from advanced diabetic cornea donors [25]. Mitochondrial function, measured using extracellular flux analysis, decreases in these tissues without a compensatory increase in glycolytic activity. Ultrastructurally, the mitochondria are distended with dense osmium staining inclusion bodies, disrupted cristae organization, and cristae drop out as visualized by transmission electron microscopy analysis. These results indicate that the mitochondrial dynamics of corneal endothelial cells are disrupted in diabetic tissues. Using morphometric analysis of the mitochondria, we have shown that the number of mitochondria does not change with disease progression, but rather the size of the mitochondria on average increases with diabetes. This indicates an overall shift in mitochondrial dynamics, leading to less functional mitochondria built up in the cell with a decreased ability to produce ATP. Overall, this would be expected to impair the operations and health of the cell, and in the worst case, lead to cell death and dropout. The high degree to which insulin dependant diabetes affects proteins associated with oxidative phosphorylation proteins, as determined in this study, strongly supports the case that chronic local hyperglycemia causes mitochondrial dysfunction in corneal endothelial cells and therefore warrants further investigations based on the pathways highlighted in this dataset (Fig 6).

Limitations of this study include depth of validation and the fact that we analyzed the entire complex rather than separating cells from Descemet membrane. We have focused primarily on the most significantly altered pathway, mitochondrial dysfunction. There were several pathways altered in this dataset (Table 3, Table 4, S3 Table) that clearly affect the health of corneal EDM tissues in T2DM. Additional investigations designed to assess the impact of changes related to oxidative phosphorylation, protein translation and quality control, organelle signaling, stress/apoptosis, and intercellular junctions are warranted to further understand the impact of T2DM on human corneal endothelial cells. Additionally, we analyzed both the basement membrane (Descemet membrane) and the endothelial cells in the same analysis. Although many proteins specific to one region can be queried in the dataset, it is impossible to determine if those proteins originated only from the matrix or only from the cells. It will be necessary to validate any claims of protein origin in the future if specificity to a single region is desired.

This research investigation provides the complete proteome of EDM tissues without disease and with different severities of T2DM. To our knowledge, it is the first dataset comparing these disease states in these tissues. It can be used as a resource for further investigations related to EDM tissues. The bioinformatics results strongly support previous findings relating diabetes mellitus and mitochondrial dysfunction as shown by our research team. These discoveries are strikingly similar to previous reports in other tissues, e.g. skeletal muscle, and provide groundbreaking support for the importance of the links between insulin resistance, mitochondrial dynamics, oxidative stress, and diabetic disease progression.

## Supporting information

S1 Table Mass spectrometry results. All peptide intensities are listed for each isotope of every protein identified in this study. Samples are identified at the top of the spreadsheep by number corresponding to demographic data in Table 1, and diagnosis is listed below each sample.
(XLS)

S2 Table ANOVA results. Multiple data sheets provided comparing all 4 groups and several different two-way comparisons including AD compared to ND, all insulin samples (NAD-i and AD) compared to non-insulin samples (NAD-ni and ND), NAD samples with and without insulin, ND compared to NAD-ni, and AD compared to NAD-i. Within the 4-way analysis, comparison fold changes are also shown for all two way comparisons (i.e. AD vs. ND), as well as each independent p-value and fold change. The second column represents the p-value for the 4-way analysis for each protein.
(XLSX)

S3 Table Pathway results. Top pathways associated with statistically significant protein changes and the complete protein lists for all pathways. Pathways were determined using Ingenuity Pathway Analysis (IPA; Qiagen, Germantown, MD).
(XLS)
